# Prurigo pigmentosa related to a popular diet trend: the ketogenic diet^☆^

**DOI:** 10.1016/j.abd.2024.08.005

**Published:** 2025-01-06

**Authors:** Cristina Barrabés-Torrella, Ana Iglesias-Plaza, María Teresa Fernández-Figueras, Montse Salleras-Redonnet

**Affiliations:** a Dermatology Department, Hospital Universitari Sagrat Cor, Grupo Hospitalario Quirónsalud, Barcelona, Spain; b Pathology Department, Hospital General de Catalunya, Grupo Hospitalario Quirónsalud, Sant Cugat del Vallès, Spain

*Dear Editor,*

Prurigo pigmentosa is a rare inflammatory disorder of unknown etiology.^1^ However, some cases have been associated with ketosis. Herein, we report a case of prurigo pigmentosa triggered by a ketogenic diet with a good response to minocycline and discontinuation of this diet.

A 30-year-old woman from Morocco, with no significant medical history, presented with pruritic lesions on her thorax, abdomen and back evolving for two weeks. The patient had erythematous, crusty and hyperpigmented papules coalescing in a reticulate pattern (Fig. 1). She reported starting a ketogenic diet a month ago, which consisted in a high-fat, adequate-protein, and low-carbohydrate diet. The cutaneous biopsy of one of the lesions described a lymphocytic dermatitis with eosinophils, acanthosis, spongiosis and neutrophilic sub-corneal pustules (Fig. 2), findings consistent with the diagnosis of prurigo pigmentosa. The patient was encouraged to stop the ketogenic diet and treated with 100 mg of minocycline a day. After a month of treatment, only post-inflammatory hyperpigmentation remained (Fig. 3).

**Fig. 1:**
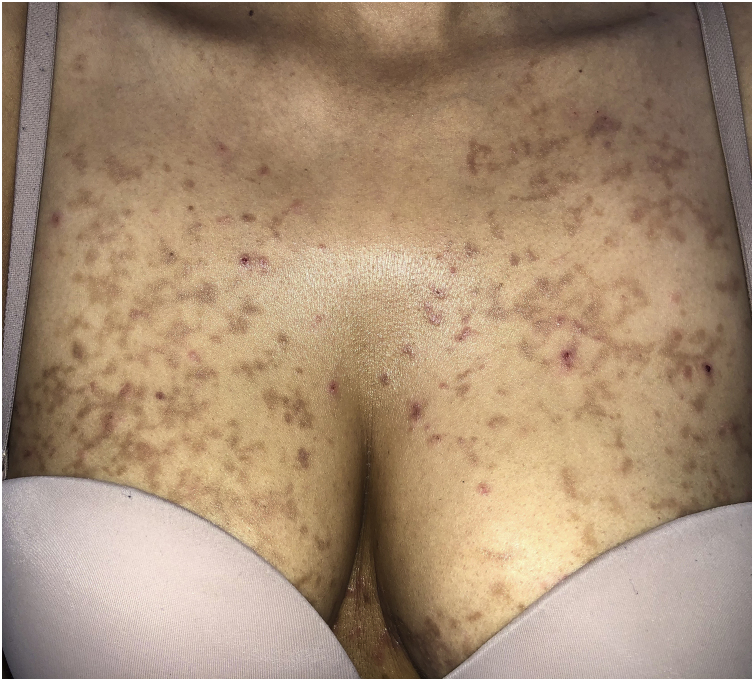
Prurigo pigmentosa: Erythematous, crusty and hyperpigmented papules in thorax.

**Fig. 2:**
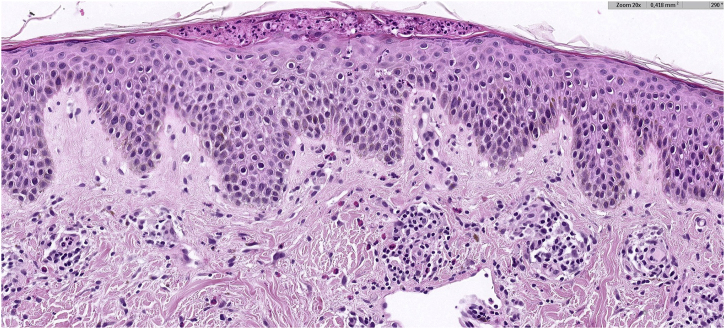
Hystopatology: Lymphocytic dermatitis with eosinophils, spongiosis, acanthosis, and a neutrophilic subcorneal pustule, findings consistent with a fully developed stage lesion of prurigo pigmentosa (Hematoxylin & eosin, ×20).

**Fig. 3:**
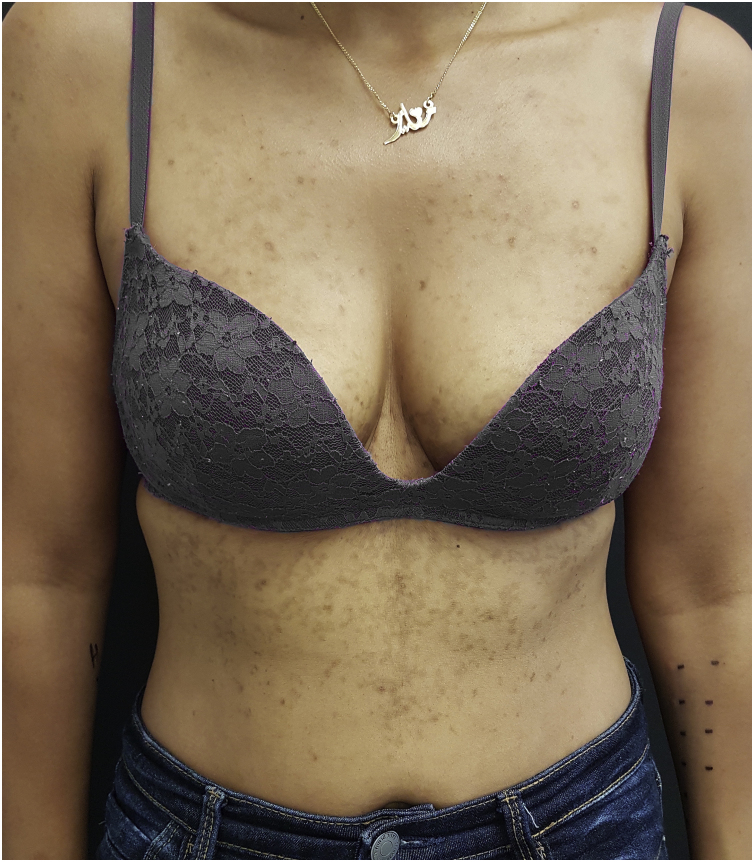
Prurigo pigmentosa: Residual hyperpigmentation after four weeks of treatment.

Prurigo pigmentosa has historically been reported in East Asian women.^1^, ^2^ The progression of the disease is typically divided into three stages: early, fully developed, and late; each with distinctive clinical and histopathological features. The disease mainly affects the back, chest, and neck. Early-stage presents with pruritic urticarial papules or plaques with an edematous papillary dermis, hyperkeratosis and a neutrophilic sparse and perivascular infiltrate in histological examination. After 2‒3 days, fully developed lesions appear, consisting of crusted erythematous papules and vesicles that correspond with the finding of hyperkeratosis, moderate spongiosis, a lymphocytic infiltrate and necrotic keratinocytes in the biopsy. These lesions usually resolve within a week, leaving hyperpigmented macules. Lesions tend to coalesce and are often found in different stages simultaneously.^3^, ^4^

Prurigo pigmentosa has a broad differential diagnosis, including reticulated papillomatosis, Dowling-Degos disease, erythema *ab igne,* or medication-induced pigmentation. The intense pruritus and the confluent reticulated pattern of the lesions are the most distinctive features of prurigo pigmentosa.

Although the etiology of prurigo pigmentosa is not well known, there may be a genetic predisposition for the development of the disease. The condition has been reported in individuals experiencing ketosis, triggered by factors such as diabetic ketoacidosis, calorie-restrictive or low-carbohydrate diets, fasting, anorexia nervosa, or bariatric surgery. The growing popularity of weight-loss diets like the ketogenic diet or intermittent fasting suggests a potential rise in the incidence of prurigo pigmentosa in the future. The ketogenic diet consists of a very low-carb, moderate protein and high-fat diet often used as a dietary regimen. The purpose of this diet is to induce a metabolic state that mimics starvation and promotes the breakdown of fat into ketone bodies as an alternative energy source.^2^ Furthermore, prurigo pigmentosa has been associated with other conditions such as atopic dermatitis, contact allergy, adult-onset Still’s disease, or Sjogren’s syndrome.^4^ In some patients, flares are triggered by hormonal changes such as pregnancy, menstruation, or polycystic ovary syndrome. Prurigo pigmentosa may also be related to bacterial infections, as one study reported an association with Helicobacter pylori gastritis^5^ and another concluded that Borrelia spirochetes may contribute to the development of prurigo pigmentosa in some patiets.^6^ Exogenous factors such as sweating, friction or contact allergens may also trigger the disease.^1^

Tetracyclines are the treatment of choice. Its efficacy is likely due to its anti-inflammatory effects and the ability to inhibit neutrophil migration. Dapsone is another effective option.^3^ In some of these patients, dietary changes alone are sufficient to treat the condition.^1^ To treat post-inflammatory pigmentation, topical hydroquinone and triple combination cream therapy (hydroquinone, retinoid and corticosteroid) could be effective, though their use may be limited by irritation. Low-dose oral tranexamic acid may be considered in patients with no risk for thromboembolic events.^7^ In recalcitrant cases of reticulate pigmentation, Q-Switched Nd:YAG laser therapy may be considered.^8^

## Financial support

None declared.

## Authors’ contributions

Cristina Barrabés Torrella: Study concept and design and writing of the manuscript.

Ana Iglesias Plaza: Effective participation in the research guidance.

María Teresa Fernández Figueras: Critical review of histopathology content and final approval of the final version of the manuscript.

Montse Salleras Redonnet: Final approval of the final version of the manuscript.

## Conflicts of interest

None declared.
